# Serum calcium levels and the risk of sarcopenia in young adults: insights from NHANES 2011–2018

**DOI:** 10.3389/fnut.2025.1526879

**Published:** 2025-03-14

**Authors:** Junliang Jiang, Ge Chen, Yonggang Li, Qinggang Zhao, Zhong Chen

**Affiliations:** Department of Orthopedics and Traumatology, Affiliated Hospital of Yunnan University, Yunnan University, Kunming, China

**Keywords:** sarcopenia, serum calcium, young and middle-aged adults, NHANES, appendicular skeletal muscle mass

## Abstract

**Background:**

Sarcopenia, the accelerated loss of muscle mass and function, is commonly associated with aging, especially in older adults. While low serum calcium has been linked to muscle loss in individuals over 50, its relationship with sarcopenia in younger adults (20–60 years) is unclear. This study examines this association using data from the National Health and Nutrition Examination Survey (NHANES) from 2011 to 2018.

**Methods:**

This population-based, cross-sectional study analyzed participants aged 20–60 from NHANES 2011–2018. Individuals with missing data were excluded. Sarcopenia was assessed using appendicular skeletal muscle (ASM) measured by dual-energy X-ray absorptiometry, defined by ASM/BMI or ASM/Weight. Multiple logistic regression and stratified analyses were used to explore the correlation between serum calcium levels and sarcopenia prevalence.

**Results:**

Among 7,309 adults, 578 (7.91%) had sarcopenia by ASM/BMI and 1,363 (18.65%) by ASM/Weight. Higher serum calcium levels were significantly associated with a lower risk of sarcopenia (ASM/BMI: OR 0.07, 95% CI 0.02–0.20; ASM/Weight: OR 0.09, 95% CI 0.04–0.19). The trend was consistent across age, gender, education, poverty income ratio, and race, with some exceptions.

**Conclusion:**

This study highlights a negative association between serum calcium and sarcopenia risk in young and middle-aged adults, suggesting that calcium interventions could be beneficial in preventing sarcopenia in this population. However, the cross-sectional design precludes any inference of causality, and further longitudinal studies are warranted to confirm these findings.

## Introduction

Sarcopenia is a condition characterized by the accelerated loss of muscle mass and function, occurring in the context of a progressive and widespread skeletal muscle disorder (1). The prevalence of sarcopenia is expected to quadruple from 50 to over 200 million cases in the next 40 years (2). This condition is linked to increased risks of mortality, fractures, hypertension, diabetes mellitus (DM), disability, and the need for hospitalization or institutionalization (1, 3–6), imposing a significant burden on healthcare finances.

Recent studies further indicate that the onset of sarcopenia is influenced by diverse factors, including demographic variables, diabetes mellitus, hypertension, and chronic kidney disease (7–10). Additionally, obesity and systemic inflammation have been implicated in exacerbating muscle deterioration and elevating metabolic risks. Although sarcopenia has traditionally been viewed as predominantly affecting older adults (1), emerging evidence shows that it can manifest earlier in life (11). For example, a notably high prevalence of sarcopenia has been documented among young and middle-aged South Korean individuals (12, 13). Moreover, hospitalization costs linked to sarcopenia are often more significant in younger adults compared to those aged 65 and older (14). Young and middle-aged adults with sarcopenia face a heightened risk of developing metabolic disorders, experiencing reduced physical function, and enduring long-term disability, highlighting the need for early intervention (15, 16). Despite this, the underlying factors contributing to sarcopenia in this population remain poorly understood and warrant further investigation. Therefore, this study focuses on identifying risk factors for sarcopenia in young and middle-aged adults (20–60 years) to develop early interventions that may reduce the long-term health impacts of muscle loss, including metabolic disorders and disability.

In young adults and middle aged adults, skeletal muscle displays higher plasticity compared to older individuals, which relies on the potential of muscle fibers to undergo structural changes and the composition of muscle protein isoforms (17). Within muscle fibers, calcium plays a pivotal role in multiple processes critical for muscle health: it not only facilitates excitation-contraction coupling by binding to troponin and regulating the actin-myosin cross-bridge cycle, but also acts as a critical signaling molecule in pathways such as mTOR and CaMK, which regulate muscle protein synthesis and degradation (18–22). Furthermore, calcium is indispensable for the proper function of calpains, which are calcium-dependent proteases involved in myogenesis and the turnover of muscle proteins (19, 21). Insufficient calcium levels may therefore disrupt these processes, leading to impaired muscle repair and regeneration, and potentially accelerating protein degradation pathways, thereby contributing to sarcopenic outcomes (23, 24). Previous studies have linked low serum calcium levels to skeletal muscle loss in older adults (≥50 years), demonstrating that reduced calcium intake predicts muscle loss (25, 26). However, limited research has examined this relationship in young and middle-aged adults.

This study collected data from NHANES 2011–2018, employing multiple logistic regression and stratified analysis to examine the correlation between serum calcium levels and the sarcopenia among adults aged 20–60 in the United States.

## Methods

### Study population

National Health and Nutrition Examination Survey (NHANES) is a cross-sectional survey conducted by the National Center for Health Statistics, which selects participants using a complex 4-stage probability sampling design. It gathers information on the health and nutritional status of the civilian, noninstitutionalized population in the United States, excluding those in correctional facilities or nursing homes. The NHANES (National Health and Nutrition Examination Survey) data utilized in this study were collected by the National Center for Health Statistics (NCHS). During data collection, informed consent was obtained from all participants, and the study protocols received approval from the NCHS Ethics Review Board. As this research involves secondary analysis of publicly available, de-identified data, it is exempt from additional ethical review or participant consent requirements.

Starting in 1999, NHANES collected and released data in 2-year cycles. Since 2007, several changes have been made to the over-sampled domains (27). Notably, the entire Hispanic population, rather than just Mexican Americans (MA), was oversampled. In addition, similar to the approach taken with those aged 60 and over in the previous cycle, Black individuals and low-income populations were oversampled to ensure accurate estimates for these specific groups (28). Due to the absence of skeletal muscle mass data between 2007 and 2010, our study selected the 2011–2012 cycle as the starting point for data inclusion. Furthermore, considering the potential unknown impacts of the COVID-19 pandemic on the data results, our research only incorporated NHANES data from 2011 to 2018.

**Figure 1:**
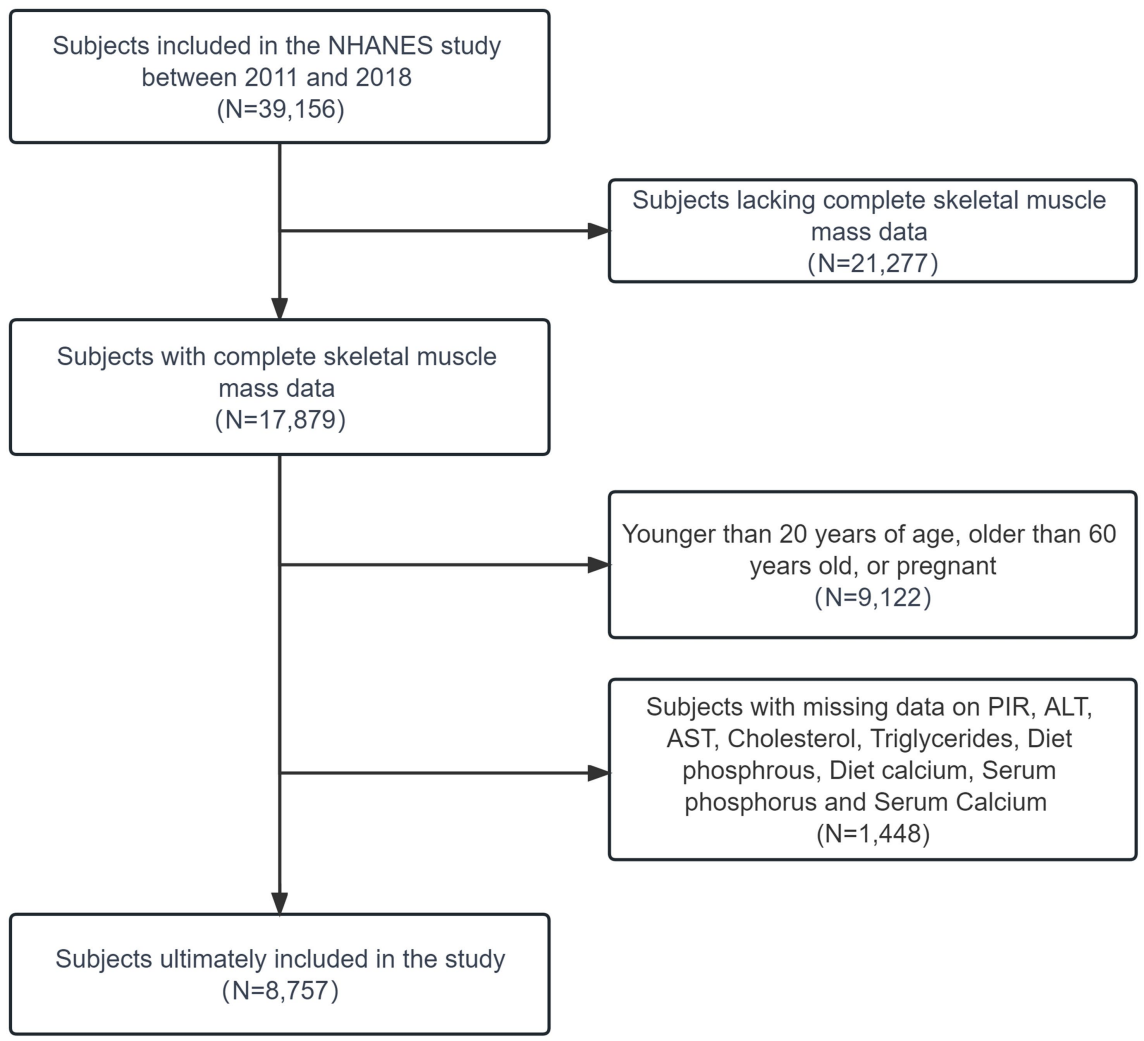
Flowchart describing sample.

Overall, between 2011 and 2018, a total of 39,156 subjects were initially included in the NHANES study. Among these, 17,879 subjects possessed complete data on skeletal muscle mass, of which 8,757 were aged 20–60 years old and were either male or non-pregnant females. Data were excluded if individuals were missing certain values, including 679 subjects lacking data on the ratio of family income to poverty (PIR), 423 subjects without data on alanine aminotransferase (ALT, U/L), and 432 subjects without data on aspartate aminotransferase (AST, U/L). Additionally, 422 subjects lacked data on cholesterol (refrigerated serum, mmol/L), 427 subjects were missing data on triglycerides (refrigerated serum, mmol/L), 470 subjects lacked data on diet calcium and diet phosphorus, 423 subjects had no data on serum phosphorus, and 439 subjects were without data on serum calcium (mmol/L). Some individuals had multiple missing data points. Ultimately, our study included 7,309 participants (Figure 1).

### Definitions of sarcopenia

The NHANES database utilized Dual-energy X-ray absorptiometry (DXA) whole-body scans to assess appendicular skeletal muscle mass (ASM) (29). DXA scan was performed using Hologic QDR 4500A fan beam X-ray bone densitometer (Hologic, Inc., Bedford, MA). Original scan results were analyzed using Hologic Discovery software (version 12.1, Hologic, Inc.) to derive fat mass and lean mass. Body regions (head, arms, trunk, and legs) were delineated manually using tools provided by the software. Trunk region was defined as the area from the inferior edge of the chin as the upper borders to the oblique lines that cross the femoral necks and converge below the pubic symphysis as the lower perimeter, with vertical borders lateral to the ribs. The area below the lower borders of the trunk was defined as leg region (30).The calculation of ASM involved summing the lean soft tissue masses of both the arms and legs. Sarcopenia lacks a universally established definition (12, 31), and research has shown that different methodologies used to define sarcopenia can significantly impact study outcomes (32).

This study adopted two distinct criteria for defining sarcopenia: 1. According to the international consensus meeting of the National Institutes of Health (FNIH) (31), sarcopenia was determined by assessing the ASM/BMI ratio, with values less than 0.789 for men and less than 0.512 for women considered indicative of sarcopenia. 2. As an alternative definition, sarcopenia was identified based on an ASM/weight (in kilograms) ratio that fell 1 standard deviation below the sex-specific mean of a younger reference group aged 20–29 years. The cutoff values for this criterion were set at less than 0.32 for men and less than 0.25 for women (33).

Although functional assessments such as grip strength and gait speed are widely used in defining sarcopenia (34, 35), they were not included in this study due to data availability constraints. Specifically, grip strength data were only collected in the NHANES 2011–2014 cycles, and gait speed was not assessed in NHANES during the study period (2011–2018). To ensure consistency across the entire dataset, we used ASM adjusted for BMI and weight as proxies for sarcopenia (31, 33). While this approach effectively evaluates muscle mass-related sarcopenia, it may not fully capture the functional aspects of the condition. Future studies incorporating functional parameters would provide a more comprehensive assessment of sarcopenia.

### Variable

In this study, our primary objective was to explore the correlation between serum calcium levels and the prevalence of sarcopenia. Given the recognized influence of dietary intake on blood calcium levels (36), we conducted a comprehensive evaluation of these components as part of our analysis. Furthermore, acknowledging the complex etiology of sarcopenia, we conducted an extensive review of the existing literature to identify several potential variables that could contribute to the occurrence of sarcopenia or calcium metabolism (29, 37–43). These variables, including Demographic Variables, DM, hypertension (HBP) and chronic kidney disease (CKD) were incorporated into our study. Detailed data collection methods for all these variables are provided below.

### Demographic variables

Age, sex, race, and education level were self-reported by participants (44, 45). BMI was calculated as a person’s weight in kilograms divided by the square of height in meters. The PIR serves as an indicator of family income in relation to the poverty threshold. It categorizes income levels into three groups: low-income (PIR ≤ 1.3), middle-income (PIR > 1.3–3.5), and high-income (PIR > 3.5) (42). Smoking status was defined based on self-report of having smoked at least 100 cigarettes in a participant’s lifetime (46). One is considered to be a drinking status if they meet any of the following criteria: 1. This includes individuals who consume at least two drinks per day for females or at least three drinks per day for males. 2. Individuals who engage in binge drinking on at least 2 days per month.3. Those with a history of daily binge drinking (47).

### DM diagnosis

A diagnosis of DM is established if any of the following conditions are met: 1. Participants who responded affirmatively to the NHANES question, “Has a doctor or other health professional ever told you that you had DM?.” 2. DM was diagnosed if participants had fasting plasma glucose levels ≥7 mmol/L (equivalent to 125 mg/dL), reported a physician diagnosis of DM. 3. Participants who are currently using medication to lower blood glucose levels (28).

### Hypertension diagnosis

Hypertension diagnosis was established using three criteria: 1. Participants who responded affirmatively to the NHANES question, “Has a doctor ever told you that you have hypertension?.” 2. Participants with SBP greater than 140 mmHg or diastolic blood pressure (DBP) higher than 90 mmHg were classified as hypertensive. If multiple blood pressure readings were available, the average reading was used for diagnosis. 3. Participants were deemed hypertensive if they were currently taking medications such as calcium channel blockers (CCBs), beta blockers, diuretics, angiotensin-converting enzyme inhibitors, and/or angiotensin II receptor blockers (ACEIs/ARBs) (48).

### Chronic kidney disease diagnosis

The diagnosis of CKD was determined based on a combination of factors, which included: 1. Participants who were informed by a doctor or health professional that they had CKD. 2. Participants with an estimated glomerular filtration rate (eGFR) of less than 60 mL/min/1.73 m^2^. 3. Participants with a randomized urinary albumin/creatinine ratio (ACR) greater than 30 mg/g. The eGFR was calculated using the Modification of Diet in Renal Disease (MDRD) study equation, represented as follows: eGFR = 175 × standardized Scr^-1.154 × age^-0.203 × 1.212 [if Black] × 0.742 [if female], Where eGFR is expressed in mL/min/1.73 m^2^ of body surface area, and Scr (serum creatinine) is expressed in mg/dL (49).

### Dietary assessment

Dietary calcium and phosphorus intake were assessed using a 24-h dietary recall interview, a well-established method for collecting detailed information on all foods and beverages consumed over the previous 24 h (50).

### Blood samples and biochemical measurements

Blood samples were collected from participants after a fasting period of more than 8 h. These samples were processed on-site and subsequently shipped on dry ice to central laboratories, where they were stored at −70°C until analysis (51). Serum calcium levels, serum phosphorus levels, ALT and AST levels, cholesterol levels in refrigerated serum, and triglyceride levels in refrigerated serum were measured.

### Statistical analyses

The study was conducted in accordance with the guidelines outlined in the Strengthening the Reporting of Observational Studies in Epidemiology (STROBE) (52).

Continuous variables were expressed as the mean (standard deviations), and categorical variables were presented as counts (percentages). Baseline characteristics among the various groups were compared using analysis of variance (ANOVA) for continuous variables and a χ2 test for categorical variables. Unadjusted and adjusted logistic regression models evaluated the relationship between serum calcium level as the primary predictor and probable sarcopenia status as the outcome. To explore potential interactions, the analysis was stratified by demographic variables. All data analyses were performed by using the Survey package in R software (version 4.3.0; R Foundation for Statistical Computing, Vienna, Austria). A two-sided *p*-value <0.05 indicated significance for all analyses.

## Result

### Characteristics of study participants

The present study analyzed data from 7,309 participants aged 20 to 60 years who participated in NHANES from 2011 to 2018 (Figure 1). The prevalence of sarcopenia calculated using the ASM/BMI method was 7.91%, and using the ASM/weight method, it was 18.65%. Baseline characteristics of participants stratified by sarcopenia status according to the ASM/BMI (Table 1) and ASM/weight (Table 2) methods revealed consistent trends across both classification approaches. Individuals with sarcopenia were older and predominantly male. Racial disparities were observed, with a higher prevalence among Mexican Americans and Other Hispanics, while Non-Hispanic Black individuals had a significantly lower prevalence. Additionally, individuals identified as sarcopenia by both methods tended to be shorter stature, higher body weight and BMI, and lower PIR. This group also showed a higher prevalence of HBP, CKD, and DM. Moreover, sarcopenic individuals exhibited elevated blood levels of ALT, AST, cholesterol, and triglycerides, along with reduced dietary phosphorus intake, dietary calcium intake, serum calcium, and serum phosphorus.

**Table 1:** Characteristics of participants according to the status of sarcopenia, defined as ASM/BMI.

Parameters	Non-sarcopenia (*N* = 6,731)	Sarcopenia (*N* = 578)	*P*
Age, years	35.91 ± 10.52	39.32 ± 11.06	<0.001
Male, *N* (%)	4,049 (60.20)	378 (65.40)	0.015
Race, *N* (%)			<0.001
Mexican American	895 (13.30)	200 (34.60)	
Other Hispanic	613 (9.10)	92 (15.90)	
Non-Hispanic White	2,527 (37.50)	157 (27.20)	
Non-Hispanic Black	1,414 (21.00)	32 (5.50)	
Non-Hispanic Asian	961 (14.30)	76 (13.10)	
Other Race	321 (4.80)	21 (3.60)	
Education, *N* (%)			<0.001
Less than high school	1,055 (15.67)	168(29.07)	
High school graduate/GED or equivalent	1,421 (21.10)	157 (27.20)	
College graduate or above	4,255 (63.21)	253 (43.77)	
PIR	2.51 (1.65)	2.06 (1.51)	<0.001
Weight, Kg	81.70 (20.43)	90.11 (26.91)	<0.001
Height, cm	170.08 (9.07)	160.82 (9.03)	<0.001
BMI, Kg/m2	28.14 (6.33)	34.47 (8.33)	<0.001
ASM	24.04 (6.37)	22.29 (6.52)	<0.001
ASM/BMI	0.87 (0.19)	0.65 (0.13)	<0.001
Smoking, *N* (%)	2,696 (40.10)	215 (37.20)	0.193
Drinking, *N* (%)	2,990 (44.40)	229 (39.60)	0.029
HBP, *N* (%)	1,572 (23.40)	198 (34.30)	<0.001
DM, *N* (%)	474 (7.00)	102 (17.60)	<0.001
CKD, *N* (%)	619 (9.20)	84 (14.50)	<0.001
ALT, U/L	26.22 (20.77)	32.94 (27.98)	<0.001
AST, U/L	25.14 (18.44)	27.31 (18.48)	0.007
Cholesterol, mmol/L	4.83 (1.02)	5.02 (1.10)	<0.001
Triglycerides, mmol/L	1.68 (1.75)	2.10 (1.80)	<0.001

Continuous variables are presented as mean ± standard deviation (SD), while categorical variables are expressed as counts and percentages. Baseline characteristics among the various groups were compared using analysis of variance (ANOVA) for continuous variables and a χ^2^ test for categorical variables. PIR refers to the Ratio of Family Income to Poverty. BMI stands for Body Mass Index. ASM/BMI represents appendicular skeletal muscle mass-to-BMI. HBP denotes Hypertension. DM refers to Diabetes Mellitus. CKD stands for chronic kidney disease. ALT represents Alanine Aminotransferase, and AST stands for Aspartate Aminotransferase.

**Table 2:** Characteristics of participants according to the status of sarcopenia, defined as ASM/Weight.

Parameters	Non-sarcopenia (*N* = 5,946)	Sarcopenia (*N* = 1,363)	*P*
Age, years	35.55 ± 10.50	38.92 ± 10.63	< 0.001
Male, *N* (%)	3,561 (59.90)	866 (63.50)	0.014
Race, *N* (%)			< 0.001
Mexican American	777 (13.10)	318 (23.30)	
Other Hispanic	560 (9.40)	145 (10.60)	
Non-Hispanic White	2054 (34.50)	630 (46.20)	
Non-Hispanic Black	1,346 (22.60)	100(7.30)	
Non-Hispanic Asian	931 (15.70)	106 (7.80)	
Other Race	278 (4.70)	64 (4.70)	
Education, *N* (%)			< 0.001
Less than high school	955 (16.06)	268 (19.66)	
High school graduate/GED or equivalent	1,234 (20.80)	344 (25.20)	
College graduate or above	3,757 (63.19)	751 (55.10)	
PIR	2.51 (1.65)	2.32 (1.61)	< 0.001
Weight, Kg	78.29(17.98)	100.15(24.44)	< 0.001
Height, cm	169.47(9.33)	168.81(9.69)	0.018
BMI, Kg/m2	27.18 (5.59)	35.01 (7.52)	< 0.001
ASM	23.65(6.26)	25(6.87)	< 0.001
ASM/Weight	0.3(0.04)	0.25(0.03)	< 0.001
Smoking, *N* (%)	2,314 (38.90)	597 (43.80)	0.001
Drinking, *n* (%)	2,632 (44.30)	587 (43.10)	0.439
HBP, *N* (%)	1,279 (21.50)	198 (36.00)	< 0.001
DM, *N* (%)	352 (5.90)	224 (16.40)	< 0.001
CKD, *N* (%)	530 (8.90)	173 (12.70)	< 0.001
ALT, U/L	25.69 (21.00)	31.36 (23.01)	< 0.001
AST, U/L	25.03 (18.66)	26.53 (17.46)	0.007
Cholesterol, mmol/L	4.81 (1.01)	4.99 (1.09)	< 0.001
Triglycerides, mmol/L	1.63 (1.71)	2.08 (1.89)	< 0.001

^Continuous variables are presented as mean ± standard deviation (SD), while categorical variables are expressed as counts and percentages. Baseline characteristics among the various groups were compared using analysis of variance (ANOVA) for continuous variables and a χ2 test for categorical variables. PIR refers to the Ratio of Family Income to Poverty. BMI stands for Body Mass Index. ASM/Weight represents Appendicular Skeletal Muscle Mass-to-Weight. HBP denotes Hypertension. DM refers to Diabetes Mellitus. CKD stands for chronic kidney disease. ALT represents Alanine Aminotransferase, and AST stands for Aspartate Aminotransferase.^

Significant differences in dietary calcium intake were noted in sarcopenic individuals identified by the ASM/BMI method, but not in those identified by the ASM/weight method. The ASM/BMI method revealed a higher proportion of individuals consuming alcohol, while the ASM/weight method showed no significant difference. Conversely, smoking was significantly more prevalent among individuals identified by the ASM/weight method, with no significant difference observed in the ASM/BMI group.

### Serum calcium is independently associated with the risk of sarcopenia

To investigate the relationship between various variables and the occurrence of sarcopenia, we conducted separate univariate logistic analyses. Under both definitions of sarcopenia, an increase in serum calcium was found to be negatively correlated with the risk of sarcopenia. Moreover, it was observed that age, gender, race, education, PIR, HBP, DM, CKD, ALT, AST, cholesterol, triglycerides, serum phosphorus, and dietary phosphorus were all identified as significant risk factors associated with the development of sarcopenia (Supplementary Tables 1, 2). After adjusting for all the covariates, it was consistently observed that higher Serum Calcium levels were associated with a decreased risk of sarcopenia (Table 3).

**Table 3:** Odds ratio of sarcopenia prevalence according to serum calcium category, sarcopenia defined by ASM/BMI and ASM/Weight.

Variable	Serum calcium	Q1	Q2	Q3	*P* trend
		OR (95% CI)	OR (95% CI)	OR (95% CI)	
Sarcopenia defined by ASM/BMI					
Model 1	0.05 (0.02–0.14)	Reference	0.69 (0.57–0.84)	0.60 (0.48–0.75)	<0.001
Model 2	0.08 (0.03–0.23)	Reference	0.74 (0.60–0.90)	0.65 (0.51–0.81)	<0.001
Model 3	0.09 (0.03–0.25)	Reference	0.75 (0.61–0.92)	0.65 (0.51–0.82)	<0.001
Model 4	0.07 (0.02–0.20)	Reference	0.73 (0.59–0.90)	0.62 (0.49–0.78)	<0.001
Sarcopenia defined by ASM/Weight					
Model 1	0.08 (0.04–0.17)	Reference	0.70 (0.61–0.81)	0.63 (0.54–0.73)	<0.001
Model 2	0.13 (0.06–0.28)	Reference	0.75 (0.65–0.86)	0.68 (0.58–0.80)	<0.001
Model 3	0.12 (0.06–0.25)	Reference	0.73 (0.64–0.85)	0.66 (0.56–0.77)	<0.001
Model 4	0.09 (0.04–0.19)	Reference	0.71 (0.62–0.83)	0.63 (0.53–0.74)	<0.001

Model 1: Not adjusted. Model 2: Adjusted by age, gender, and race. Model 3: Further adjusted for education level, smoking status, alcohol consumption, hypertension (HBP), diabetes mellitus (DM), chronic kidney disease (CKD), and the Ratio of Family Income to Poverty (PIR). Model 4: Additionally adjusted for alanine aminotransferase (ALT), aspartate aminotransferase (AST), cholesterol, triglycerides, serum phosphorus, serum calcium, dietary phosphorus, and dietary calcium. ASM/BMI, Appendicular Skeletal Muscle Mass-to-Body Mass Index; ASM/Weight, Appendicular Skeletal Muscle Mass-to-Weight. Q1, Q2, and Q3 represent tertiles of serum calcium levels.

**Figure 2:**
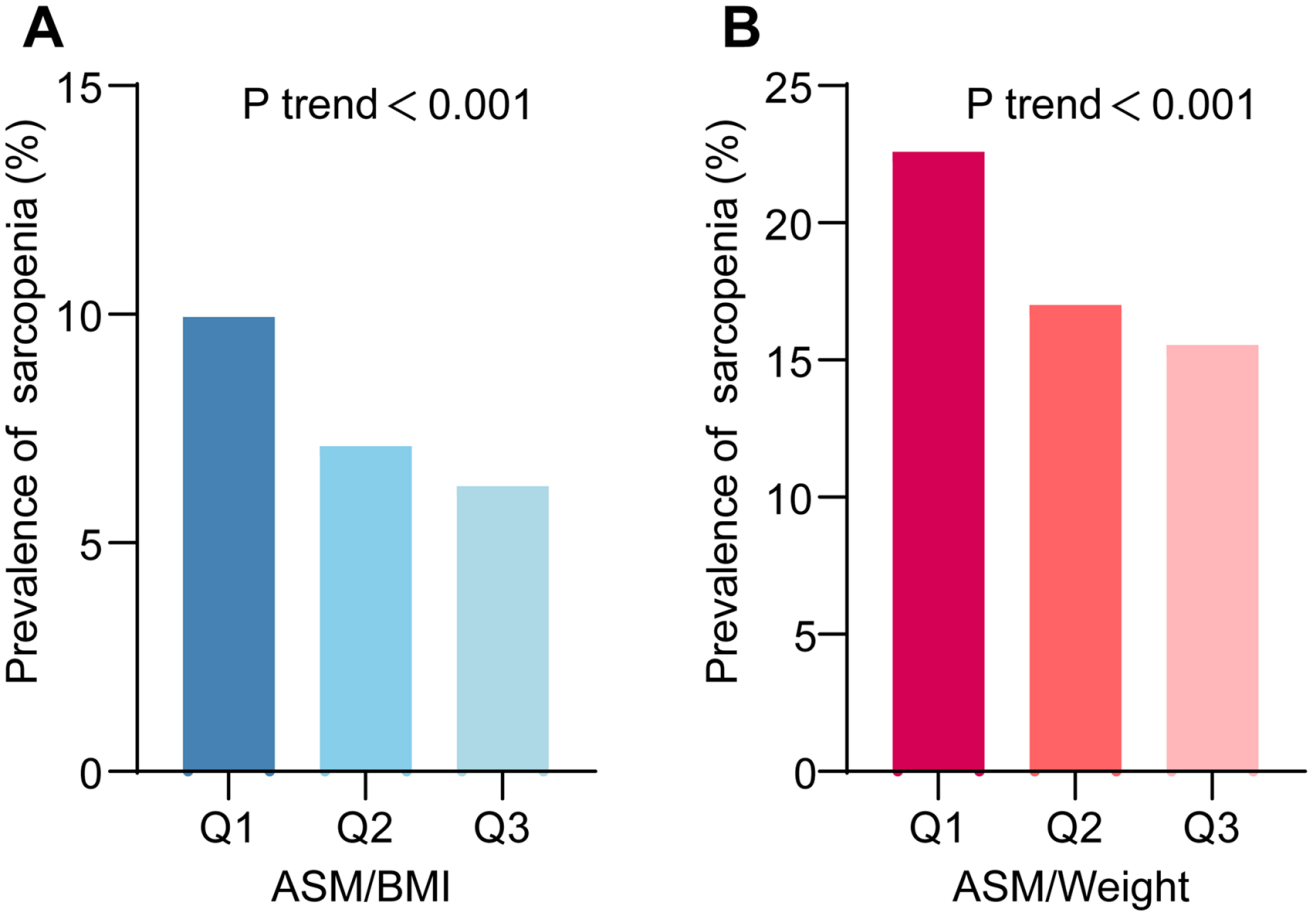
The prevalence of sarcopenia across different levels of serum calcium. **(A)** Sarcopenia defined using the appendicular skeletal muscle mass-to-body mass index (ASM/BMI) method. **(B)** Sarcopenia defined using the ASM-to-Weight (ASM/Weight) method. Q1, Q2, and Q3 represent tertiles of serum calcium levels.

To further elucidate the association between Serum Calcium and sarcopenia, we stratified Serum Calcium values into three tiers (Q1, Q2 and Q3). The result revealed that with increasing Serum Calcium levels, there was a gradual dose–response reduction in the risk of sarcopenia. Importantly, this trend remained consistent across different diagnostic criteria for sarcopenia (Figures 2A,B).

### Subgroup analysis

**Figure 3:**
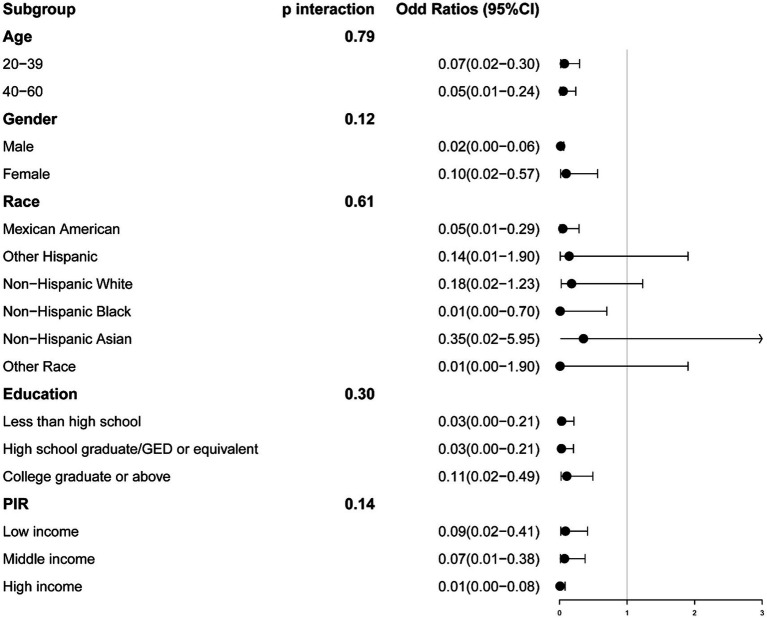
The association between serum calcium and sarcopenia within selected subgroups, with sarcopenia defined by appendicular skeletal muscle mass-to-body mass index (ASM/BMI). PIR refers to the Ratio of Family Income to Poverty.

**Figure 4:**
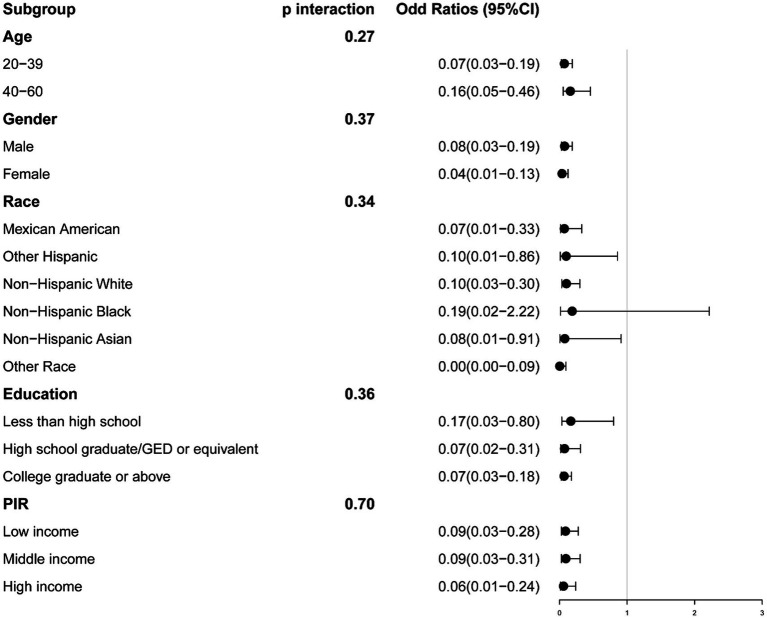
The association between serum calcium and sarcopenia within selected subgroups, with sarcopenia defined by appendicular skeletal muscle mass-to-weight (ASM/Weight). PIR refers to the Ratio of Family Income to Poverty.

We conducted stratification based on age, gender, race, education, and PIR. No significant interactions were identified between serum calcium and these stratifying variables. The relationship between serum calcium and the risk of sarcopenia remained consistent, except for specific racial groups (Sarcopenia defined by ASM/BMI: Other Hispanic, Non-Hispanic White, Non-Hispanic Asian, and Other Race; Sarcopenia defined by ASM/Weight, Non-Hispanic Black) (Figures 3, 4).

## Discussion

This study demonstrates a robust inverse association between serum calcium levels and sarcopenia risk in young and middle-aged adults (ages 20–60), irrespective of the definition criteria used. These associations persisted even after adjusting for potential confounding factors. Moreover, the observed dose–response trend across serum calcium tertiles further supports this association. Importantly, stratified analyses by demographic factors confirmed the consistency of this relationship, highlighting the potential relevance of serum calcium as a biomarker for sarcopenia risk assessment in this age group.

Sarcopenia’s prevalence estimates vary significantly due to differences in diagnostic criteria and cut-off thresholds. For instance, an NHANES-based study defining sarcopenia by ASM/BMI reported prevalences of 4.25% (ages 18–39) and 8.85% (ages 40–64), with variations by ethnicity and sex (53). Meanwhile, a Korea National Health and Nutrition Examination Survey analysis, using ASM/Weight, found prevalences of 19.2 and 29.1% in the 20–39 and 40–64 age groups, respectively (54). In the large-scale Kangbuk Samsung Health Study (over 300,000 participants), sarcopenia was identified via skeletal muscle mass index; using −1 SD and − 2 SD from a younger reference group (ages 20–39), prevalence ranged from 12.7–26.7% in females and 10.3–13% in males at −1 SD, and 2.4–5.7% in females and 1.7–2.9% in males at −2 SD (13). Consistent with these epidemiological patterns, our findings showed a sarcopenia prevalence of 7.91% (ASM/BMI) and 18.65% (ASM/Weight) in adults aged 20–60, highlighting the importance of early prevention strategies in younger populations.

Notably, discrepancies in dietary calcium intake were observed among sarcopenic individuals when classified by ASM/BMI versus ASM/weight. This inconsistency may arise from differences in how each method accounts for body composition. ASM/BMI focuses on muscle mass relative to body size, while ASM/Weight considers total body weight, which can be influenced by factors like adiposity. Increased body fat may impact calcium absorption and metabolism, as well as alter dietary habits, potentially leading to variations in calcium intake and metabolism (55–58). Additionally, altered hormonal regulation due to higher adiposity, such as changes in insulin sensitivity or vitamin D metabolism, may also play a role in modulating calcium dynamics in the body (59, 60). Given these interactions, variations in body fat distribution may partly explain the inconsistencies observed in dietary calcium intake across different sarcopenia classification methods.

Furthermore, racial disparities in sarcopenia prevalence were evident in our findings. Consistent with prior NHANES data (38), our study revealed that sarcopenia prevalence was significantly higher among Mexican Americans and Other Hispanics, whereas Non-Hispanic Black individuals exhibited a markedly lower prevalence. Several factors may contribute to these differences. Genetic predispositions influencing muscle mass and bone density likely play a role, as Non-Hispanic Black individuals typically have greater skeletal muscle mass and bone mineral density (61–64), which may confer resistance to sarcopenia. Additionally, disparities in lifestyle factors—including physical activity levels, dietary patterns, socioeconomic conditions, and healthcare accessibility—could further influence sarcopenia risk across racial and ethnic groups (61–63, 65, 66). These findings underscore the importance of considering racial and ethnic heterogeneity when developing targeted sarcopenia prevention strategies.

Previous research suggests that dietary and metabolic factors influence sarcopenia risk, with higher energy intake and metabolic syndrome components linked to increased susceptibility (12, 54, 67). Moreover, elevated levels of glycated hemoglobin and subclinical hyperthyroidism have been correlated to a higher risk of sarcopenia in individuals with type 2 DM (68, 69). Conversely, adequate protein intake (70, 71), sufficient vitamin D levels (72), and the administration of *Lactobacillus plantarum* probiotics (73) have demonstrated an association with a reduced risk. In this study, multiple logistic regression analysis revealed that a significant inverse relationship between serum calcium levels and sarcopenia risk (ASM/BMI: OR 0.07, 95% CI 0.02–0.20; ASM/Weight: OR 0.09, 95% CI 0.04–0.19). Specifically, each unit increase in serum calcium corresponded to a 93–95% reduction in sarcopenia likelihood. These findings align with calcium’s well-established physiological roles in muscle function. For instance, Calcium ions (Ca^2+^) regulate excitation–contraction coupling and act as signaling molecules in critical pathways such as CaMK and calcineurin, thereby influencing muscle growth and fiber type composition (18, 22). Moreover, appropriate mitochondrial Ca^2+^ uptake is essential for ATP production, although excessive Ca^2+^ can lead to mitochondrial dysfunction and muscle atrophy (74). Consequently, maintaining calcium homeostasis is crucial for preserving muscle integrity and preventing sarcopenic progression.

Subgroup analyses confirmed that this inverse association remained significant even after controlling for variables potentially affecting calcium metabolism (PIR, DM, CKD, HBP, and serum phosphorus). While most demographic factors did not modify this relationship, certain racial groups showed distinct patterns. These observations corroborate earlier findings that documented substantial racial and ethnic differences in sarcopenia prevalence and characteristics (38), potentially reflecting variations in body composition, genetic factors, dietary habits, physical activity levels, and broader socioeconomic factors (62, 65, 75–78). Future research should thus incorporate racial and ethnic diversity to clarify the underlying mechanisms and explore how differing sarcopenia definitions impact these populations.

Several limitations should be noted in this study. First, cross-sectional design restricts causal inferences, making it challenging to determine whether low serum calcium levels are a cause or consequence of sarcopenia. Second, due to limited grip strength data in NHANES, our study diagnosed sarcopenia based solely on muscle mass without considering grip strength. Third, while NHANES collected physical activity data during the 2011–2014 cycles, the absence of this information in subsequent cycles prevented us from adjusting for physical activity levels, a critical confounder in muscle health studies. Fourth, dietary calcium intake was assessed using a single 24-h dietary recall, which may not accurately reflect habitual intake and is subject to recall bias (79).

Fifth, some participants lacked data on key variables in this study. These missing values have likely resulted from participants not providing the necessary information or not undergoing specific examinations. To address this, we employed listwise deletion, excluding any records with missing values. However, this approach can reduce statistical power and may introduce selection bias, potentially affecting the generalizability of our findings. Future studies should consider advanced statistical methods, such as multiple imputation, to handle missing data more effectively and minimize potential biases. Finally, our subgroup analysis indicated that many factors related to serum calcium were not sources of heterogeneity in its association with sarcopenia, underscoring the need for more empirical evidence to elucidate how serum calcium influences sarcopenia development. Therefore, further prospective studies are warranted to validate these findings.

## Conclusion

In summary, this study identified a significant inverse association between serum calcium levels and sarcopenia risk in young and middle-aged adults, consistent across demographic subgroups. These findings highlight the potential role of calcium in muscle health, suggesting that adequate dietary calcium intake and supplementation may help prevent sarcopenia. Given the cross-sectional design, future longitudinal studies are needed to establish causality and assess the effectiveness of calcium-based interventions.

## Data Availability

The original contributions presented in the study are included in the article/Supplementary material, further inquiries can be directed to the corresponding authors.
