# Supplementary material for: Serum calcium levels and the risk of sarcopenia in young adults: insights from NHANES 2011–2018
Source: Front Nutr. 2025 Mar 14;12:1526879. doi: 10.3389/fnut.2025.1526879 (PMC11951309; doi:10.3389/fnut.2025.1526879)
Supplement: Supplementary file 1 [file Table_1.docx]

**Supplementary Table 1**. Crude odds ratios (ORs) with 95% confidence intervals (CIs) of sarcopenia, defined by **ASM/BMI**.

^Abbreviation: HBP for hypertension, DM for diabetes mellitus, CKD for chronic kidney disease, PIR for poverty income ratio, ALT for alanine aminotransferase and AST for aspartate aminotransferase.^

**Supplementary Table 2**. Crude odds ratios (ORs) with 95% confidence intervals (CIs) of sarcopenia, defined by **ASM/Weight**.

_Abbreviation: HBP for hypertension, DM for diabetes mellitus, CKD for chronic kidney disease, PIR for poverty income ratio, ALT for alanine aminotransferase and AST for aspartate aminotransferase._
